# Bridging the AI chasm in oncology: a standardized platform to enable the *in silico* clinical validation of AI models within the CHAIMELEON Project

**DOI:** 10.1186/s41747-026-00770-7

**Published:** 2026-07-31

**Authors:** Adrian Galiana-Bordera, Javier Aquerreta-Escribano, Pedro Miguel Martinez-Girones, Pau Lozano, Gloria Ribas, Paula Jimenez-Gomez, J. Damián Segrelles Quilis, Leonor Cerda-Alberich, Ignacio Blanquer-Espert, Luis Marti-Bonmati

**Affiliations:** 1 Biomedical Imaging Research Group (GIBI230), La Fe Health Research Institute (IIS La Fe), València, Spain; 2 Computing Science Department, Universitat Politècnica de València, València, Spain; 3 La Fe Medical Imaging Department, Valencia, Spain

**Keywords:** Artificial intelligence, Diagnostic imaging, Medical oncology, Software, Validation studies as topic

## Abstract

**Objective:**

Artificial intelligence (AI) shows promise for improving cancer management, but clinical adoption is limited by the absence of standardized validation tools. As an important step toward bridging the AI chasm, the European CHAIMELEON project addresses this gap by developing a platform for *in silico* validation of AI models in oncology, aligned with the EUCAIM project.

**Materials and methods:**

We designed a web-based platform using Kubernetes microservices architecture, integrating imaging, clinical, and AI components. The backend combines a REST API, an ORTHANC-PACS, and a Keycloak/OAuth2 security layer. The frontend includes a customized OHIF DICOM viewer for oncological case review. Clinicians evaluated cases across three sequential stages: (1) Standard clinical assessment, (2) Assessment with AI predictions, and (3) Final review against clinical endpoint ground truth. The platform also collects clinicians’ perceptions of AI utility, trust, workflow integration, and usability through a Likert-scale questionnaire.

**Results:**

The platform provided efficient access to multimodal data for five cancer types. Clinicians completed structured validations, and survey responses indicated favorable perceptions: 93% found the platform intuitive, over 80% would recommend it, and agreement on AI utility exceeded 40% across most endpoints, indicating a positive reception of the AI as a supportive “second reader” tool that prompts clinical re-evaluation rather than simple consensus. Workflow impact was rated positively, underlining the potential to support clinical decision-making.

**Conclusion:**

This platform offers a reproducible, scalable, and user-friendly environment for clinical validation of AI models in oncology. Its modular architecture allows integration of additional AI models and is designed to ensure sustainability and interoperability, fostering evidence-based AI adoption in European radiology practice.

**Trial registration:**

ClinicalTrials.gov, NCT06950996.

**Key Points:**

- How can the medical community standardize the *in silico* clinical validation of oncology AI models to safely bridge the gap before real-world deployment?
- We successfully implemented a web-based microservices platform, demonstrating high clinical usability and defining human-AI trust dynamics across five distinct cancer types.
- This standardized validation framework directly enhances diagnostic confidence and interdisciplinary medical collaboration, bridging technical performance with clinical trust to ensure that innovative artificial intelligence technologies safely optimize daily treatment decisions and ultimately maximize therapeutic success and safety for oncology patients.

**Graphical Abstract:**

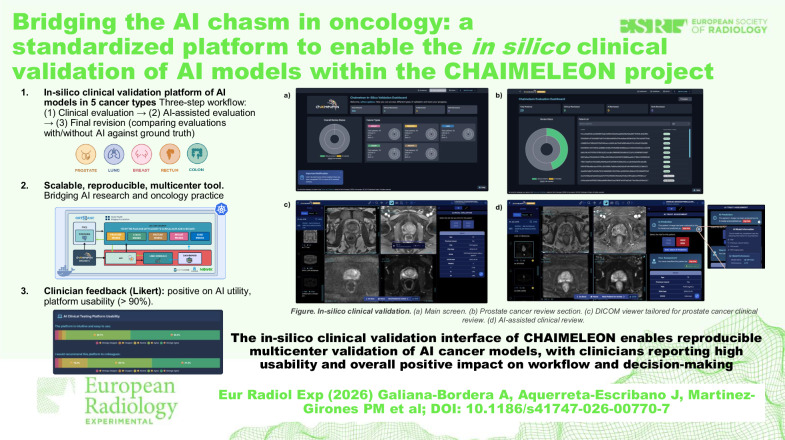

## Background

Contemporary oncology is undergoing a transformation driven by the integration of advanced technologies, particularly artificial intelligence (AI). AI has demonstrated significant potential to enhance cancer care by supporting earlier detection, tumor characterization, predicting patient outcomes, and personalizing treatment strategies [1–4]. However, despite the proliferation of high-performance algorithms in research settings, their translation into daily clinical practice remains limited, as underlined by recent position and survey papers on AI integration and validation in radiology [5, 6]. This gap is called the “AI chasm,” and it is attributable to fragmented data ecosystems, heterogeneous validation practices, and insufficient reproducibility of results across institutions, scanner vendors, and acquisition protocols. Issues that have been repeatedly highlighted in methodological radiomics studies and reproducibility analyses [7–9].

To address these barriers, European initiatives such as CHAIMELEON and the EUCAIM project have established federated infrastructures and pan-European repositories [1–3]. These efforts, supported by the AI for Health Imaging (AI4HI) network, provide the essential foundations for interoperable, GDPR compliant access to high-quality oncologic data, enabling multicenter studies and reproducible AI benchmarking at scale [8, 10–14]. Furthermore, the effectiveness and robustness of the specific pseudonymization profiles applied to these datasets have been validated through community-driven re-identification challenges, proving the security of the infrastructures against privacy leaks [15]. Within this framework, an open challenge was launched through grand-challenge.org to engage the global community in developing predictive models for five cancer types, with the final deployment and testing conducted securely within the CHAIMELEON ecosystem [1]. However, existing tools often focus on different stages of the AI lifecycle. While benchmarking platforms like Grand-Challenge prioritize algorithmic performance through leaderboards, and frameworks such as MONAI offer robust libraries for model development and deployment, they generally lack a dedicated environment for clinical end-user evaluation. The proposed platform distinguishes itself by providing an *in silico* clinical validation workspace that prioritizes the interaction between human and AI. Unlike technical benchmarking tools, this system implements a structured three-stage workflow to assess not only technical accuracy but also the clinical utility, trust, and workflow integration of AI models as ‘second readers’ in a realistic practice setting.

**Fig. 1:**
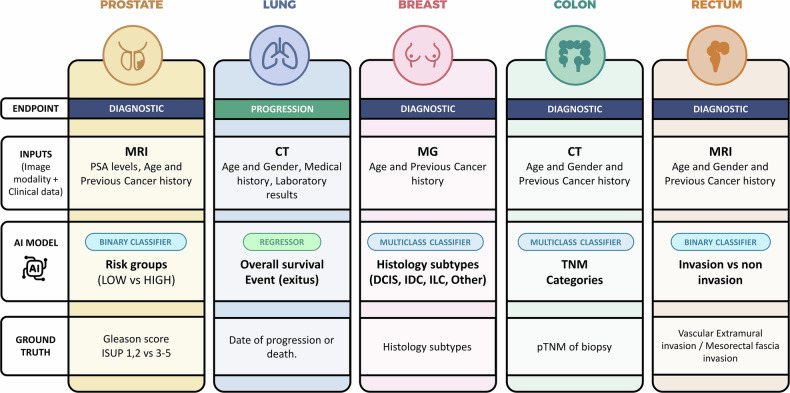
Clinical endpoints and AI models developed for the five cancer types in the CHAIMELEON project. The diagram illustrates the specific predictive tasks: Gleason score risk for prostate (low *versus* high), overall survival for lung cancer, histological subtype for breast, pTNM staging for colon, and rectum invasions

Five winning models were selected, one for each cancer type, by integrating specific imaging modalities with clinical data such as age, gender, and medical history. For prostate cancer, a binary classifier was developed using Magnetic Resonance Imaging (MRI) and PSA levels to differentiate between low and high-risk groups based on Gleason scores or ISUP (International Society of Urological Pathology). This was followed by lung cancer, where a regressor model utilized CT and medical history, treatments, and laboratory results to predict overall survival. In the case of breast cancer, a multiclass classifier was trained on mammography (MG) to identify various histological subtypes, including DCIS, IDC, ILC, or others. Finally, for colorectal malignancies, the study employed a multiclass classifier for colon cancer to categorize TNM staging from CT scans and a binary classifier for rectal cancer to detect vascular extramural or mesorectal fascia invasion through MRI (Fig. 1). Validating such models under conditions that closely simulate daily practice is essential to assess their utility, trustworthiness, and impact on clinical workflows, a need that is also emphasized by recent work on independent validation of deep learning tools and radiomic feature robustness.

International guidelines highlight the need for trustworthy AI, stressing transparency, reproducibility, human oversight, and continuous monitoring as prerequisites for safe deployment in healthcare. Within medical imaging, methodological rigor in radiomics and quantitative imaging has been reinforced through initiatives such as the Image Biomarker Standardisation Initiative (IBSI) and quality scoring systems for radiomics studies, as well as consensus recommendations from professional societies, which collectively emphasize harmonization of feature extraction, validation, and reporting [7, 9, 16–19]. Recent work has also exposed gaps between technical and clinical perspectives on AI validation in cancer imaging. It is underscoring the importance of aligning performance metrics, robustness checks, and usability criteria across disciplines. Together, these advances provide the scientific basis for reproducible AI research but also point to the need for practical tools that can evaluate AI models *in situ*, within realistic clinical workflows.

The present study addresses this gap by developing a web-based platform for *in silico* clinical validation of AI models in oncology, simulating clinical practice. The platform integrates imaging, clinical, and AI predictions in a modular environment, allowing clinicians to evaluate cancer cases with and without AI, alongside conventional imaging review and ground truth endpoints, by using data flows compatible with emerging European cancer imaging infrastructures [2, 3, 8, 13]. The web interface embedded usability and trust assessments derived from current guidelines and validation frameworks. This approach enables reproducible, scalable, and user-centered validation, fostering evidence-based adoption of AI in oncologic imaging and supporting the transition from proof-of-concept algorithms to robust clinical decision support tools [5, 6, 16, 19–21].

## Methods

### *In silico* validation pipeline overview

**Fig. 2:**
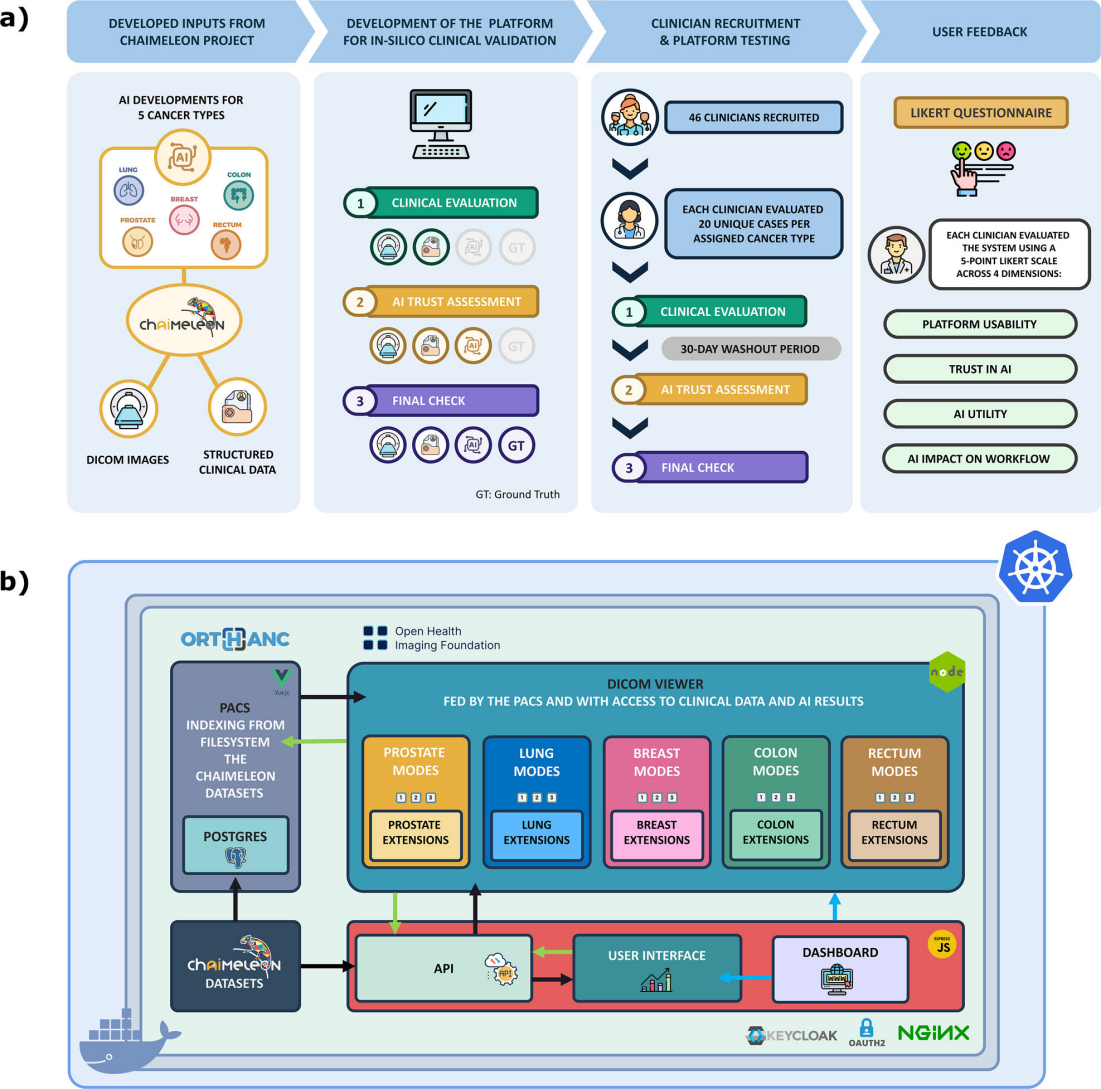
Methodological pipeline and system architecture. **a** Detailed flowchart of the *in silico* validation process: integration of data and AI models, clinician recruitment, the sequential evaluation stages, and user feedback. **b** Microservices architecture diagram detailing the separation of concerns between the Node.js backend (REST API, Keycloak Security, ORTHANC PACS) and the frontend web application

The study follows a comprehensive *in silico* validation pipeline structured into four main phases, as illustrated in Fig. 2a:

1. **Input integration:** Integration of DICOM images and structured clinical data from the CHAIMELEON project with five pre-developed AI models.
2. **Platform implementation:** Deployment of a microservices-based environment designed to support a multi-user clinical review (Fig. 2b).
3. **Clinical validation:** A structured three-stage evaluation conducted by a multicenter cohort of specialists.
4. **Qualitative assessment:** Collection of user feedback across four dimensions of perception (usability, trust, utility, and workflow impact) *via* Likert scales.

### Integrated AI models

The platform integrated five distinct AI models developed within the CHAIMELEON ecosystem: a binary classifier for prostate risk stratification, a regressor for lung cancer survival prediction, and multiclass classifiers for breast histology, colon TNM staging, and rectal vascular invasion. These models utilize multimodal inputs, including MRI, CT, or MG imaging combined with specific clinical variables such as age, PSA levels, or laboratory results (Fig. 1).

### Clinician recruitment and participation

**Fig. 3:**
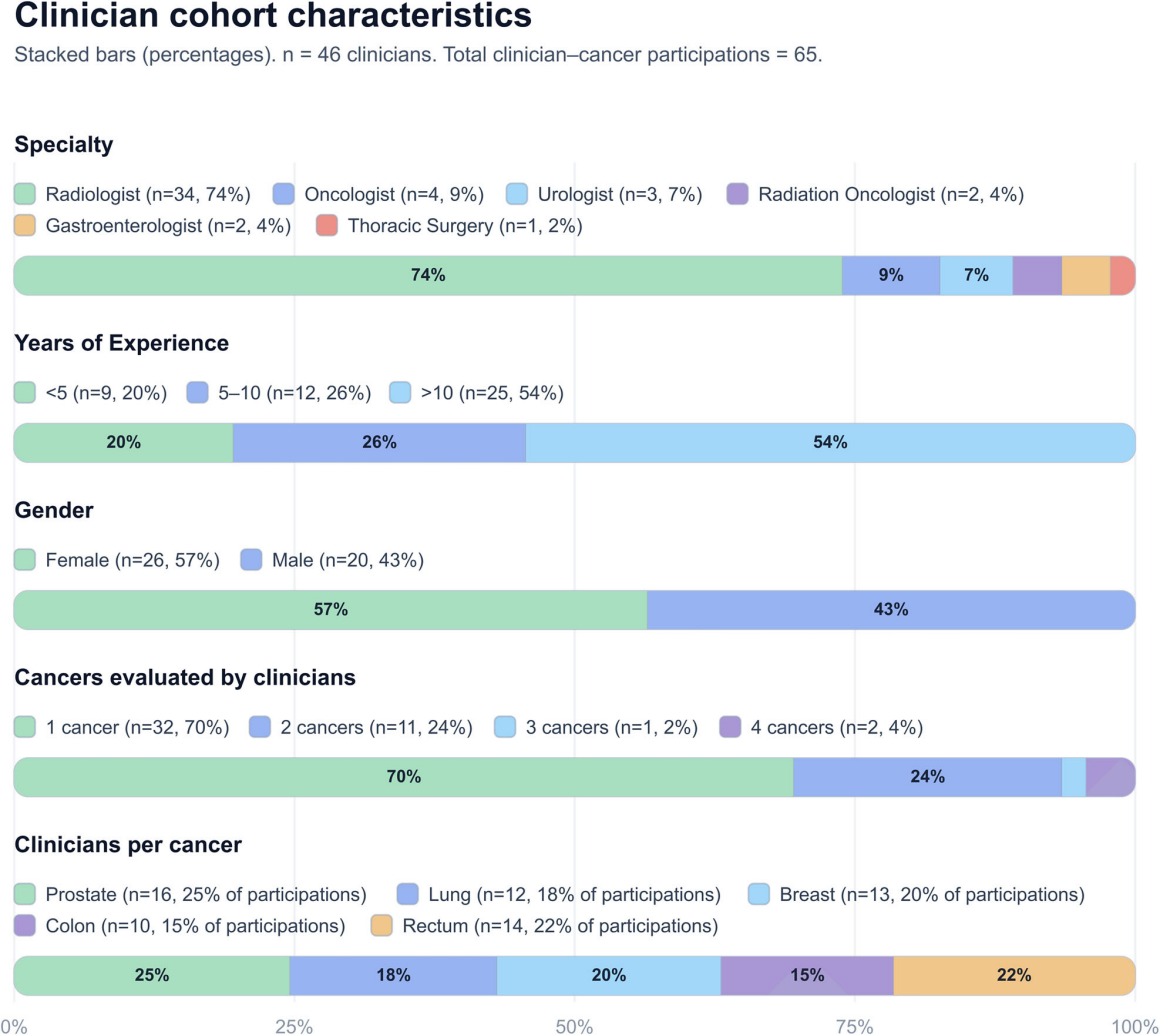
Clinician cohort characteristics and clinician-cancer participation distribution

Aligning with the principles of *In Silico* Clinical Trials (ISCT) [22], our pipeline utilizes a virtual cohort of 300 unique retrospective cases to assess AI performance as a medical device through a focused Verification and Validation (V&V) approach. A total of 46 unique clinicians were recruited from the CHAIMELEON consortium *via* targeted invitations to participate in this feasibility assessment. Each clinician was assigned 20 unique cases for each cancer type evaluated, resulting in 1,320 individual clinical assessments across standardized stages. This expert cohort (*n* = 46) was predominantly composed of radiologists (74%), oncologists (9%), and urologists (7%), with 54% of participants reporting more than 10 years of clinical experience. While 30% of clinicians evaluated more than one cancer type, we collected 65 distinct clinician-cancer participations: prostate (*n* = 16), rectum (*n* = 14), breast (*n* = 13), lung (*n* = 12), and colon (*n* = 10), as detailed in Fig. 3. The sample size (*n* = 46) was determined by seeking representative expert coverage for all five cancer types. According to literature on medical device usability, a cohort of 15 to 20 evaluators per specific category is typically sufficient to identify the majority of usability issues [23]. Our distribution (ranging from 10 to 16 clinicians per cancer) aligns with these feasibility standards for multicenter pilot assessments. This virtual environment ensures the credibility required to support future prospective deployment, and the study’s transparency is guaranteed through its registration at ClinicalTrials.gov (NCT06950996).

The clinical validation process is categorized as follows:

1. Standard clinical evaluation: Clinicians are provided with only the patient´s medical images and associated clinical data. Based on this information, they must predict the biopsy result or event without assistance from AI models.
2. AI trust assessment: In this case, clinicians review the case with the same data as in the previous step, but with the addition of the AI prediction.
3. Qualitative final review: Clinicians are given access to the results of biopsy or clinical event endpoints, along with the evaluations they made with and without the AI model. This stage allows them to determine if the AI contributed to improved diagnostic accuracy or faster decision-making.

To mitigate recall bias, a washout period of 30 days was enforced between the completion of the standard clinical evaluation and the start of the AI-assisted phase. This interval ensured that clinicians could not rely on their memory of specific cases during the subsequent AI trust assessment.

The system architecture was designed prioritizing four fundamental aspects: efficiency in data processing, security in handling sensitive medical information, scalability to adapt to increasing volumes of data, and usability catering to the user experience of clinicians. This last aspect had materialized through an intuitive and efficient user interface, specifically designed to facilitate the work of medical professionals in validating AI tools.

### System architecture

The platform was built following a microservices architecture, clearly separating backend and frontend functionalities for greater flexibility in system development and maintenance (Fig. 2b) in Kubernetes.

#### Backend

1. **REST API:** A REST API was implemented using Express.js, a Node.js framework. This API acts as the core of the system, managing the application logic and facilitating communication between different components. The REST API allows clinical data and user profile management; processing of reviews conducted by clinicians; generation and labeling of data subsets to later associate them with clinicians for evaluation; and implementation of AI model validation logic.
2. **Medical image storage and management system:** For efficient handling of radiological images, a Picture Archiving and Communication System (PACS) was implemented using the open-source ORTHANC PACS [24]. This system was integrated with a PostgreSQL database to index and manage images stored in the CHAIMELEON cloud. This configuration allows for quick and efficient access to large volumes of medical images, as well as advanced indexing to facilitate searches and retrieval of specific studies without information duplication.
3. **Security layer:** System security was ensured through the implementation of Keycloak, an identity and access management system, in conjunction with the OAuth2 protocol [25, 26]. This security layer provides robust user authentication; granular role-based authorization, where clinicians who perform the validation have a clinical-staff role; protection of sensitive API endpoints; and compliance with security standards in medical data handling.
4. **Custom interfaces in the DICOM viewer:** Extensions in Node.js were developed to expand the system’s functionality related to medical image visualization. These extensions allow real-time linking of medical images with their corresponding clinical data and AI model results. Additionally, user interaction interfaces through buttons or selectors indicate the patient evaluation (with or without AI assistance).

#### Frontend

1. **Web application:** The user interface was developed as a web application using Express.js in combination with frontend technologies. This application provides an intuitive and responsive interface for clinical users that allows clear visualization of all patients to be reviewed, access to review counters, as well as access to clinical data and AI model results.
2. **DICOM viewer**: The OHIF (Open Health Imaging Foundation) open-source DICOM viewer was integrated to provide image visualization capabilities with multiplanar reconstructions, 3D rendering, measurement, and annotation tools [27]. OHIF is “extensible” through Node.js, allowing the creation of new extensions that integrate with the viewer.

This deployment strategy provided high availability and fault tolerance, horizontal scalability to handle increases in workload, and ease of updating and maintaining individual components. This architecture provides a robust, secure, and scalable platform for clinical validation of AI models in oncology, facilitating collaboration between clinical professionals and researchers in the field of artificial intelligence applied to medicine, providing a good user experience.

### Likert questionnaire

To evaluate the usability of the *in silico* clinical validation platform and the clinical utility of its embedded models, a quantitative assessment was conducted *via* a structured Likert-scale questionnaire. Specific Likert dimensions were prioritized over general frameworks such as the System Usability Scale (SUS) [28] to better capture the unique trust and explainability requirements of oncological AI validation. Participants recorded their level of agreement on a scale of five points ranging from 1 (strongly disagree) to 5 (strongly agree). This methodology was designed to quantify clinician perspectives across four primary dimensions: platform usability, AI utility, trust in the AI, and the impact on clinical workflow. By measuring five distinct levels of agreement, the survey provided a framework to assess platform intuitiveness, perceived accuracy, and the practical integration of recommendations driven by AI into the medical routine. Likert scales were employed to transform qualitative professional perceptions into quantitative data, a method widely validated for evaluating satisfaction and workflow integration in clinical informatics [29, 30]. This allows for the identification of specific trust gaps (*e.g*., explainability) that simple binary ‘yes/no’ questions would fail to capture.

The assessment of platform usability specifically focused on whether the interface was intuitive and the likelihood of professional recommendation to colleagues. Regarding AI utility, the survey evaluated whether model predictions aligned with clinical judgment and if the selected endpoints measured meaningful patient outcomes. To ensure clarity and clinical relevance across specialized contexts, these questions were tailored to specific medical scenarios. This section also examined the capacity of the system to prioritize urgent cases and its contribution to diagnostic consistency across different healthcare settings.

Furthermore, the methodology used in this study explored the amount of trust clinicians demonstrated in the system as well as the impact on their professional workflow. This included assessing the perceived reliability of the models, the clarity of the findings for patient communication, and whether clinicians felt the need to re-evaluate patient data when AI outputs diverged from their own judgment. The survey also measured the efficiency of the platform, specifically whether the AI facilitated more rapid diagnosis and reporting compared to traditional methods.

## Results

### Platform deployment and technical performance

The *in silico* clinical validation platform was successfully deployed as a cloud-based solution, fully integrated within the CHAIMELEON infrastructure. Accessibility was established *via* a secure subdomain (https://in-silico-validation.chaimeleon-eu.i3m.upv.es/), ensuring centralized access for multicenter validation. The implementation of the microservices architecture utilizing Docker containers on Kubernetes demonstrated robust scalability, efficiently handling the simultaneous orchestration of five distinct cancer datasets (prostate, lung, breast, colon, and rectum).

The backend performance, supported by the ORTHANC PACS system and the PostgreSQL database, ensured rapid retrieval of DICOM images and associated clinical metadata. The Keycloak identity management layer, operating *via* the OAuth 2.0 protocol, successfully enforced role-based access control, ensuring that clinicians accessed only the anonymized patient cohorts relevant to their specialty (*e.g*., radiologists, oncologists, surgeons) while maintaining strict compliance with data protection standards.

### User interface and validation workflow

The customized frontend, built upon the OHIF framework, provided a responsive environment for the three-stage validation process.

**Fig. 4:**
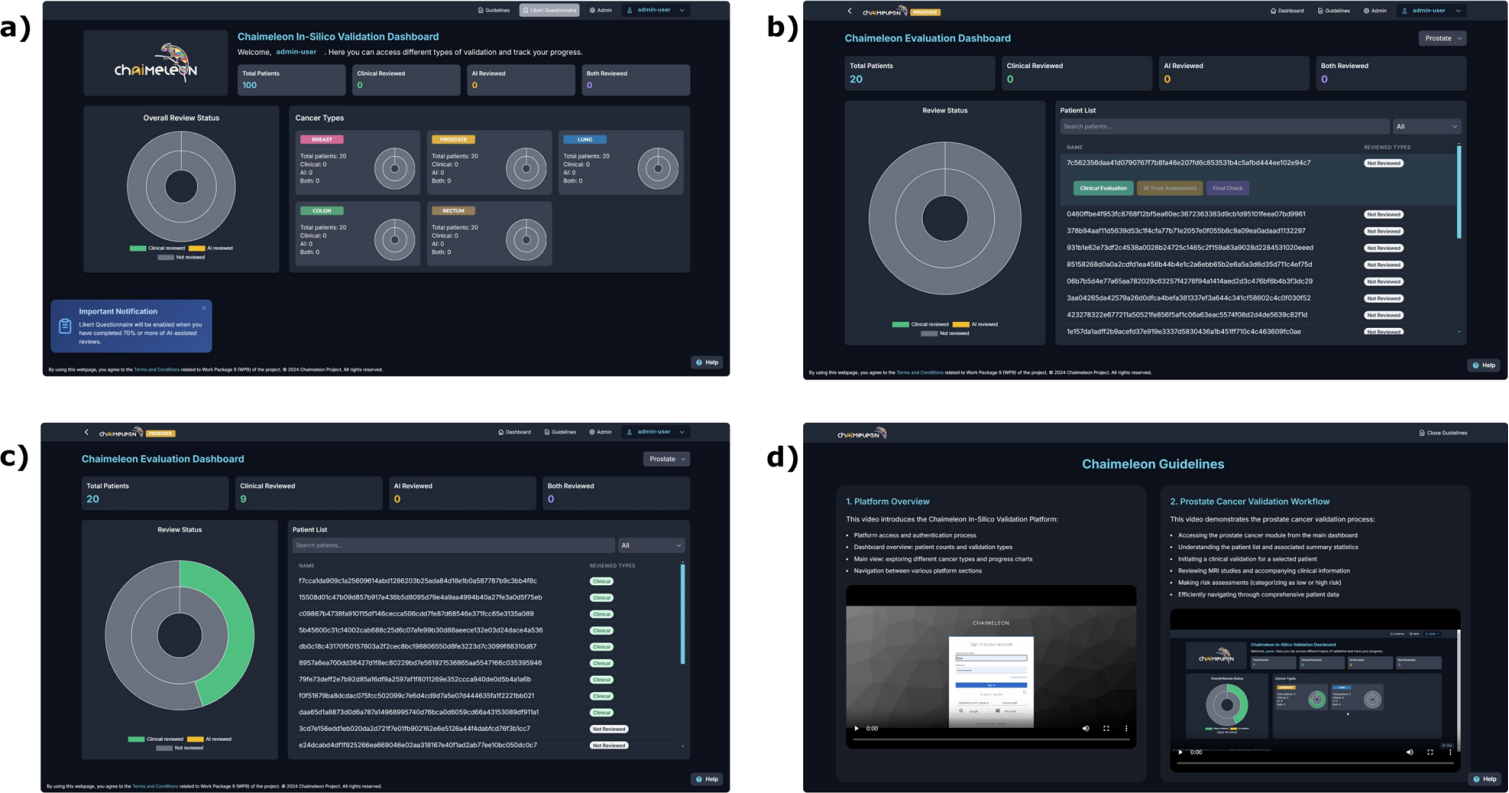
Clinician dashboard and study management interface. **a** Main user dashboard displaying the five active cancer cohorts available for validation. **b** Patient worklist view for Prostate cancer, showing validation status indicators. **c** Real-time progress charts providing visual feedback on the completion rates of the validation phases. **d** Guidelines and instructional videos offering in-depth guidance on how to perform validations

Case management: Upon login, the web interface provided a dashboard to clinicians with a clear overview of assigned cancer cohorts and real-time progress metrics (Fig. 4a). The patient selection interface (Fig. 4b) allowed for seamless navigation through the validation list of anonymized patients, with visual indicators for completed and pending cases. Upon their initial access to the validation platform, clinicians are restricted to ‘Step 1: Standard Clinical Evaluation,’ with subsequent stages remaining locked until the baseline assessment is complete. Once the evaluation is performed, the system updates to display the ‘Clinical’ label and reflects the user’s progress within the tracking plot (Fig. 4c). To facilitate ease of use, the platform includes integrated guidelines that comprise video tutorials, accessible to clinicians for clarification on system functionality (Fig. 4d).

**Fig. 5:**
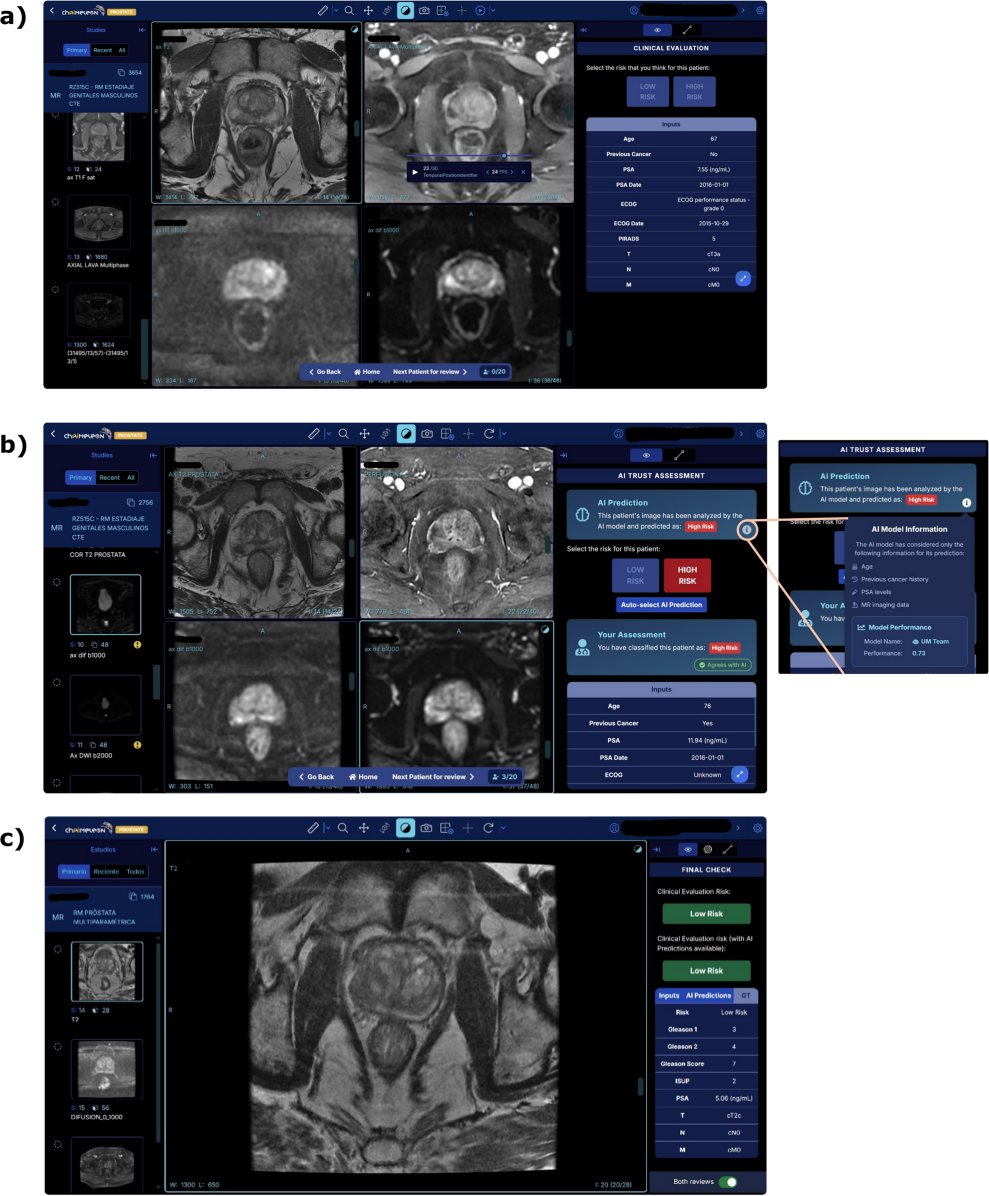
The three-stage *in silico* validation interface using the customized OHIF Viewer. **a** Stage 1: standard clinical evaluation, where the clinician reviews the MRI/CT images and clinical data without AI support. **b** Stage 2: AI trust assessment, displaying the AI model’s prediction (*e.g*., risk stratification) alongside the images to evaluate its influence on the clinician’s decision. **c** Stage 3: final review, unlocking the ground truth for comparative analysis against the human and AI-assisted predictions

Sequential Evaluation: The platform successfully enforced the sequential logic of the validation study to prevent bias:

1. **Standard clinical evaluation:** In the initial stage, clinicians utilized a customized DICOM viewer to analyze radiological images and clinical variables without AI assistance (Fig. 5a). Upon completing 100% of the assigned cases, this baseline evaluation mode was locked to prevent further modifications.
2. **AI trust assessment:** Following a washout period of 30 days after the completion of the Phase 1 standard clinical evaluation, the AI-assisted evaluation mode was unlocked. In this interface, the AI model’s predictions, such as prostate cancer risk, are overlaid onto the clinical data (Fig. 5b). Clinicians are presented with a prediction card that includes an information icon providing details on model performance and the underlying logic of the AI generation. To streamline the workflow, an auto-selection feature allows users to quickly adopt the AI-suggested classification. Finally, a summary card displays the clinician’s assessment (*e.g*., low *versus* high risk in prostate cancer) and explicitly indicates the agreement or disagreement between the human evaluator and the AI model.
3. **Qualitative final review:** The final stage involves unlocking the ground truth, which includes biopsy results or clinical outcome data. This phase enables a comprehensive comparative review where clinicians assess the concordance between their initial baseline diagnosis, the AI prediction, and the definitive clinical gold standard (Fig. 5c).

This sequential unlocking mechanism was implemented to maintain strict data isolation between stages. By preventing the premature exposure of information, the platform ensured the integrity of the comparative analysis between independent clinical judgment and AI-assisted evaluations.

#### Clinician perception and platform usability

**Fig. 6:**
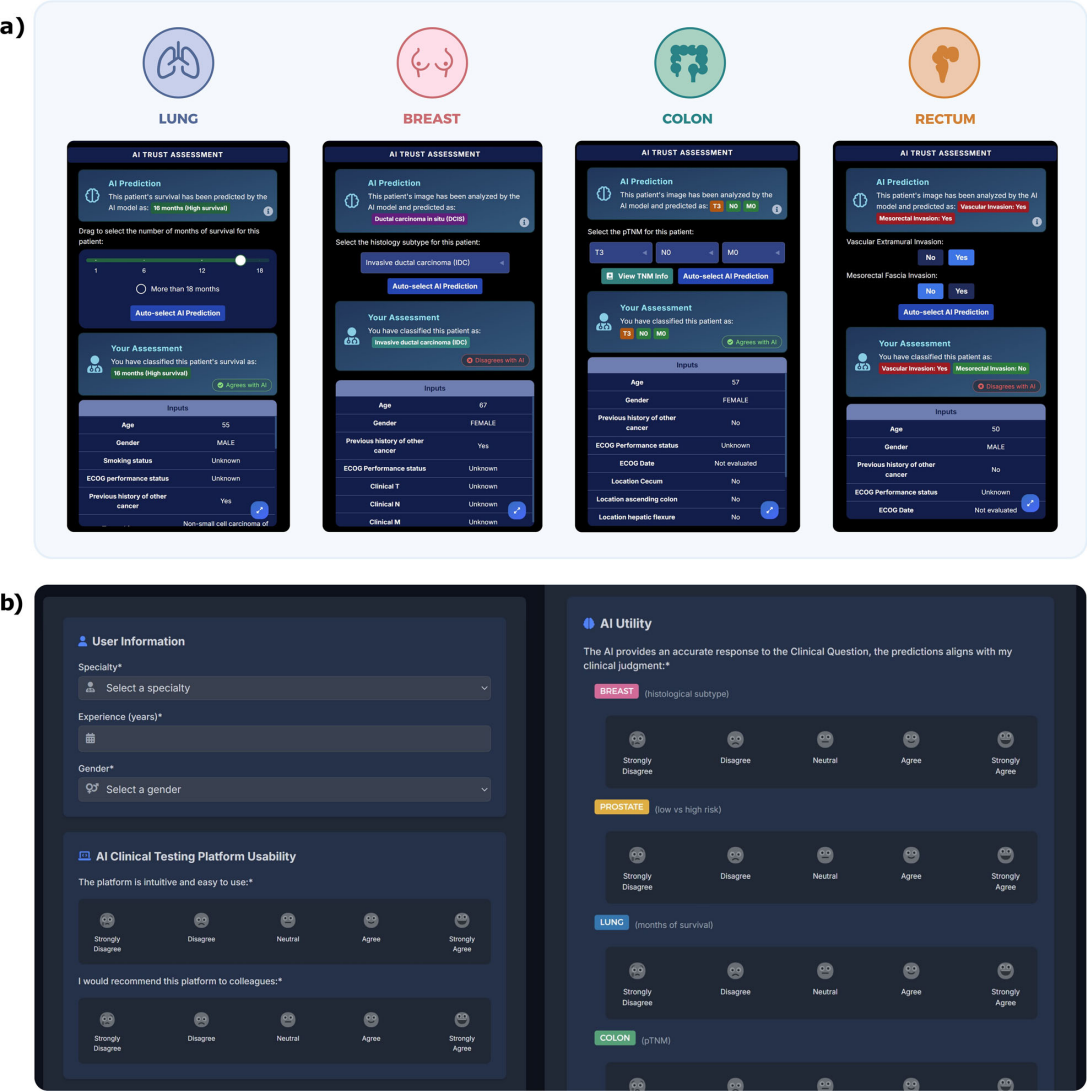
AI trust assessment interface and feedback mechanism. **a** Assessment panels for lung, breast, colon, and rectum cancers. **b** Visual interface of the Likert-scale questionnaire used for clinician feedback

Following validation across five cancer types (prostate, lung, breast, colon, and rectum), Fig. 6a illustrates the remaining stages of the AI Trust Assessment. The interface options were tailored to each malignancy to ensure the user experience aligned with the specific clinical context, as further detailed in Supplementary Fig. 1 in the Supplementary Material. To evaluate this experience, clinician feedback was collected through a Likert-scale questionnaire integrated directly into the platform (Fig. 6b). As shown, the questionnaire spans from strong disagreement to strong agreement and utilizes a visual design with emojis and a red-to-green color gradient to enhance respondent engagement. Overall, the results indicate high acceptance of the system and a positive perceived utility of the AI-assisted workflow.

### Usability and adoption

**Fig. 7:**
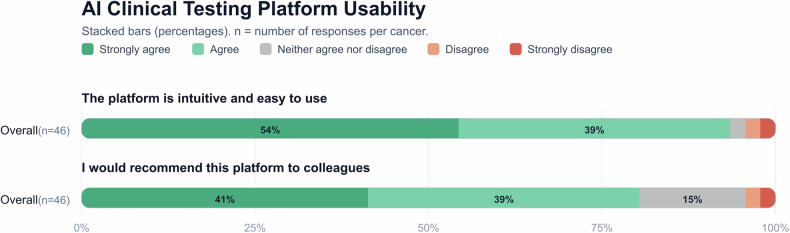
Platform usability and adoption. Stacked Likert-scale distributions summarizing clinician responses regarding platform usability (intuitiveness/ease of use) and adoption intent (recommendation to colleagues). Values are shown as percentages of responses; n denotes the number of respondents

The platform achieved high usability ratings. Specifically, 93% of participating clinicians (*n* = 46) rated the platform as intuitive and easy to use. Participants frequently attributed this to the familiar OHIF-based viewer layout, which is similar to a standard clinical PACS workstation. Furthermore, 80% of respondents (*n* = 46) expressed a willingness to recommend the platform to colleagues for AI validation tasks (Fig. 7).

### AI utility and trust

Quantitative analysis of survey responses revealed differences in perceived utility and trust depending on the clinical endpoint and cancer type.

**Fig. 8:**
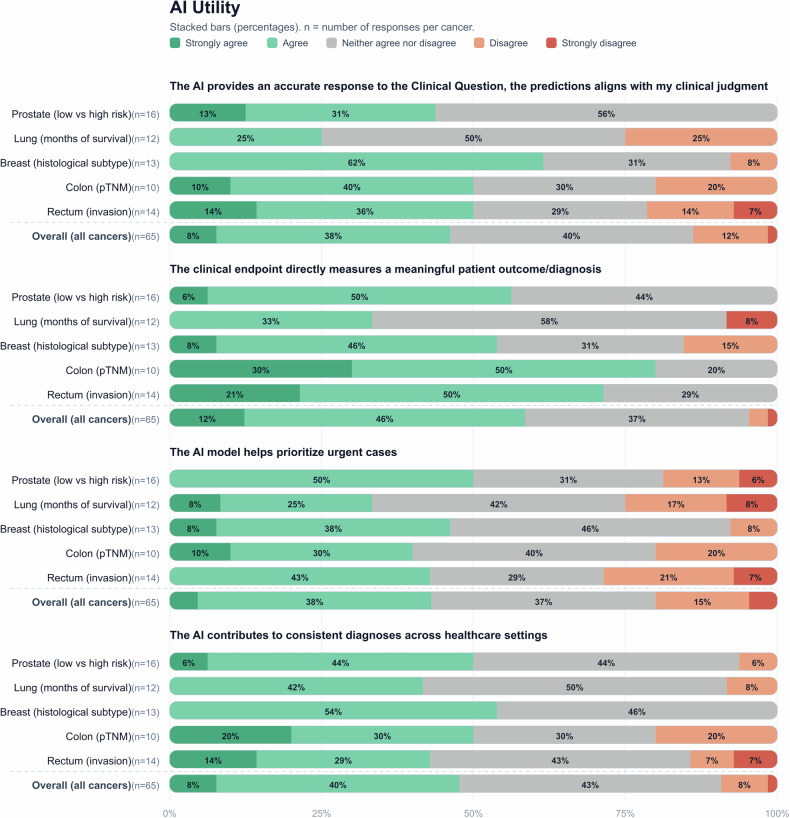
Perceived AI utility by clinical endpoint and cancer type. Stacked Likert-scale distributions for perceived AI utility statements (accuracy alignment with clinical judgment; meaningfulness of the clinical endpoint; prioritization of urgent cases; contribution to diagnostic consistency), stratified by cancer type, with an overall pooled summary row where applicable. Values are shown as percentages; n denotes the number of responses per cancer (and pooled overall)

**Fig. 9:**
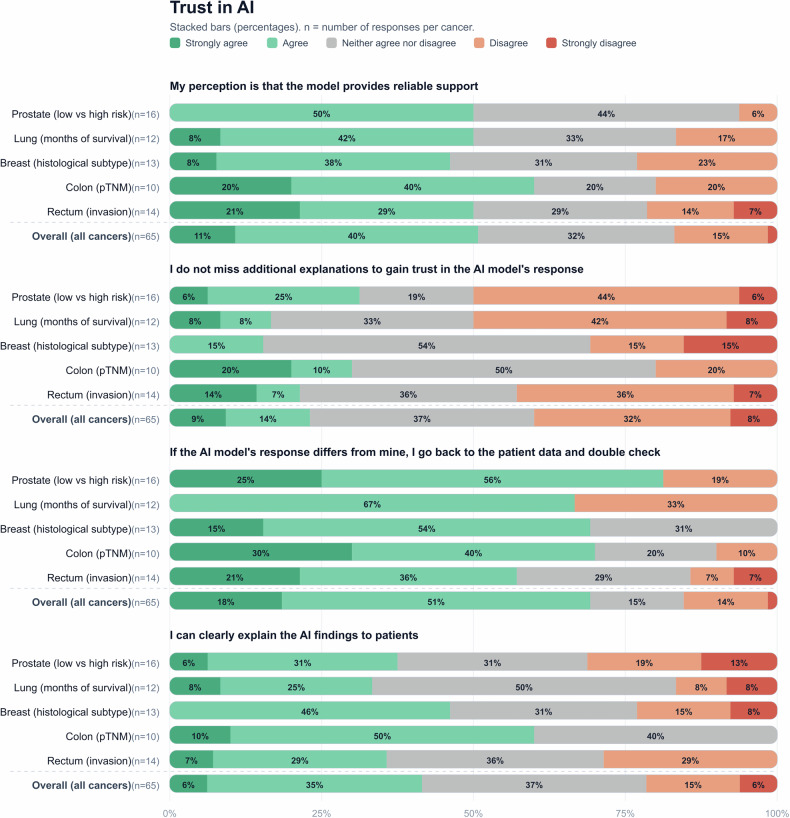
Trust in AI, verification behavior, and explainability. Stacked Likert-scale distributions for trust-related statements (perceived reliability of AI support; need for additional explanations; tendency to double check patient data when discordant; ability to explain AI findings to patients), stratified by cancer type, with an overall pooled summary row where applicable. Values are shown as percentages; n denotes the number of responses per cancer (and pooled overall)

- **Perceived utility across endpoints**: Overall, 58% of responses were favorable (Agree/Strongly agree, *n* = 65) for the statement “The clinical endpoint directly measures a meaningful patient outcome/diagnosis” (Fig. 8). Perceived utility was highest for colorectal tasks, with 80% agreement in colon cancer (*n* = 10) and 71% in rectal cancer (*n* = 14). In contrast, perceived utility was lower for lung cancer survival prediction (33%, *n* = 12), consistent with the increased complexity of prognostication compared with diagnostic/staging tasks.
- **Perceived accuracy alignment**: For the statement “The AI provides an accurate response to the Clinical Question, and the prediction aligns with my clinical judgment,” favorable responses were 46% overall (*n* = 65), with lower agreement for lung cancer (25%, *n* = 12) than for breast (62%, *n* = 13), colon (50%, *n* = 10) or rectum (50%, *n* = 14) (Fig. 8).
- **Trust and verification behavior**: Notably, 69% of responses (*n* = 65) agreed with “If the AI model’s response differs from mine, I go back to the patient data and double check.” This supports the interpretation of the platform as a second reader mechanism that prompts re-evaluation of discordant cases. In addition, 51% of responses (*n* = 65) agreed that “the model provides reliable support” (Fig. 9).

### Workflow integration and explainability

**Fig. 10:**
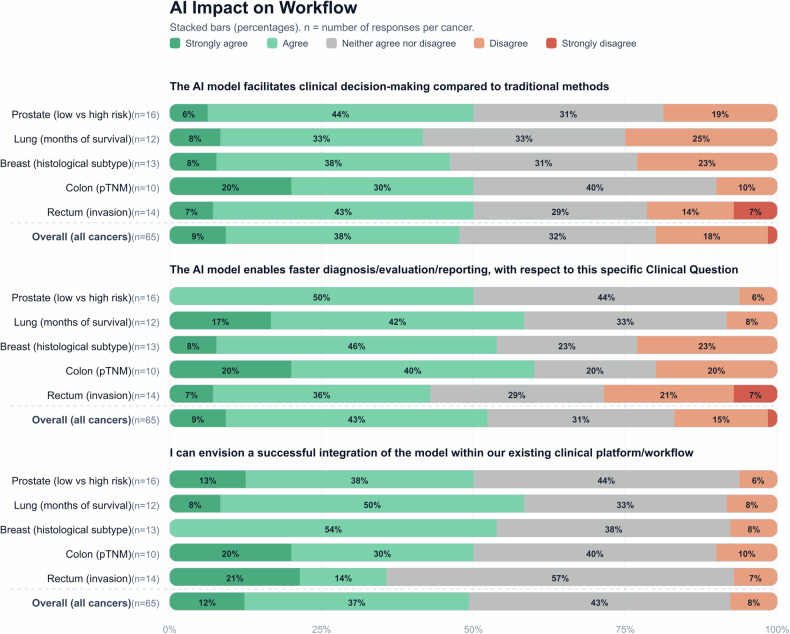
AI impact on workflow and integration readiness. Stacked Likert-scale distributions for workflow impact statements (facilitating clinical decision-making; enabling faster diagnosis/evaluation/reporting; feasibility of integration into existing workflows), stratified by cancer type, with an overall pooled summary row where applicable. Values are shown as percentages; n denotes the number of responses per cancer (and pooled overall)

Workflow impact was perceived positively but highlighted a persistent explainability gap (Fig. 9 and Fig. 10).

- **Workflow integration**: 49% of responses (*n* = 65) agreed with “I can envision a successful integration of the model within our existing clinical platform/workflow.” Related items were also rated favorably, including facilitating clinical decision-making (48%, *n* = 65) and enabling faster diagnosis/evaluation/reporting (52%, *n* = 65) (Fig. 10).
- **Explainability gap and patient communication**: Only 41% of responses (*n* = 65) agreed with “I can clearly explain the AI findings to patients.” Moreover, only 23% of responses (*n* = 65) agreed with “I do not miss additional explanations to gain trust in the AI model’s response.” These findings suggest that, while the platform is effective in presenting results and supporting review, future iterations should incorporate stronger interpretability features (*e.g*., saliency maps, feature importance summaries, or case-based rationales) to bridge trust and communication needs (Fig. 9).

## Discussion

### A paradigm shift from algorithmic precision to clinical utility

The current landscape of oncology reflects a technological paradox where the exponential growth of artificial intelligence models contrasts with their limited adoption in daily clinical routine. This gap, described as the AI chasm, suggests that the primary barrier to implementation is not a lack of technical performance but the absence of standardized and user-centered validation environments.

The web platform developed under the CHAIMELEON project addresses this critical gap by creating an *in silico* clinical trial (ISCT) environment that replicates the oncological workflow. Aligning with the framework proposed by Pathmanathan et al [22], this study utilizes a virtual cohort to provide a high degree of credibility during the verification and validation (V&V) process. By facilitating the validation of predictive models across five distinct pathologies (prostate, lung, breast, colon, and rectal cancer), this study provides empirical evidence that technical validation must be accompanied by usability, utility, and trust validation. The widespread acceptance of the platform by clinicians, with over 93% rating it as intuitive and over 80% willing to recommend it, reinforces the potential of ISCTs to bridge the AI chasm.

The Sequential Evaluation Methodology employed in this study, which is structured into (1) standard clinical evaluation, (2) AI trust assessment, and (3) final qualitative review, marks a substantial shift from traditional “black box” validation. This three-stage workflow serves as a robust V&V framework, allowing us to effectively decouple baseline clinical performance from AI-assisted outcomes. This distinction allowed us to quantify not only diagnostic accuracy but also the tangible value the tool adds to clinical confidence and decision-making. In this framework, the finding that agreement on AI utility exceeded 40% across most endpoints provides an initial baseline for future comparative systems. To date, specific literature benchmarks establishing typical clinician agreement or perceived utility levels for multi-cancer *in silico* validation platforms do not yet exist in the field. Therefore, this 40% threshold serves as an early empirical anchor rather than an absolute milestone. Rather than seeking a perfect consensus that might render the AI redundant, this level of agreement suggests the models provide a complementary perspective that prompts deeper clinical reflection.

The fact that 69% of clinicians re-verified patient data suggests the tool functions as an effective second read. This three-stage workflow reveals the diagnostic trajectory by logging how a clinician moves from an unassisted baseline to an AI-informed decision. By capturing these transitions, the platform facilitates a measurable audit of clinical re-evaluation, reinforcing the synergy between clinician intuition and algorithmic support while breaking potential automation bias. This architectural capability ensures that any diagnostic shift is registered, providing a transparent record of how AI influences the final clinical outcome.

### Architectural foundations for European federated research

The sustainability and scalability of AI validation platforms depend on their architectural decisions. This study validates a microservices-based approach orchestrated *via* Kubernetes, integrating mature open-source technologies such as ORTHANC PACS for image management and OHIF for advanced visualization. This choice is not merely a technical preference but a strategic alignment with the FAIR principles (Findable, Accessible, Interoperable, and Reusable) that govern the European Health Data Space and the EUCAIM initiative.

Unlike traditional PACS systems, which often exhibit rigidity and significant vendor lock-in, a container-based architecture provides flexibility. This modularity allows for the independent updating of critical components. For example, if a new AI model for prostate cancer requires specific or diverse computational capacity, the inference service can scale horizontally without compromising the performance of the DICOM viewer or the latency of the underlying PostgreSQL database.

Furthermore, this architecture prepares the platform for the future of federated research. In the context of the EUCAIM project, which aims to federate over 60 million oncological images by 2026 [2], the ability to deploy validation logic on local nodes within hospitals is fundamental. Since the CHAIMELEON *in silico* validation platform is containerized, it acts as a distributed validation node that allows code to travel to the data. This approach of bringing code to data minimizes the privacy risks and GDPR compliance issues typically associated with the cross-border transfer of sensitive patient data. The platform will be integrated into EUCAIM, facilitating future AI validation within the project.

### Security, identity management, and workflow integration

Security in the handling of medical data is a non-negotiable requirement for clinical adoption. The implementation of Keycloak alongside the OAuth2 protocol provides a robust and granular security layer that manages user authentication and authorization with precision. In multicenter validation studies involving diverse professional profiles such as radiologists, oncologists, surgeons, and data researchers, the ability to define specific roles and restrict access to subsets of anonymized data is critical.

The successful integration of these security protocols demonstrates that it is possible to balance strict data protection with the user experience.

### Human-centric visualization and the explainability gap

The customization of the OHIF (Open Health Imaging Foundation) viewer constitutes one of the central innovations of this work. Commercial PACS viewers are optimized for rapid diagnosis but often lack the flexibility necessary to display experimental AI overlays or collect structured feedback in real-time. Conversely, many basic research viewers lack the multiplanar reconstruction (MPR) and 3D rendering tools that radiologists require for accurate clinical assessments.

By extending the OHIF framework using Node.js, we have created a hybrid environment that maintains familiar clinical tools while introducing research functionalities. These include buttons for clinical validation, information on guidelines for the cancer under review, and AI predictions, all designed to be easy to use. As our findings from the Likert questionnaire suggest, clinicians place higher trust in local explanations that point to specific image features compared to global explanations or abstract confidence metrics.

However, our analysis of clinical confidence also reveals a notable “trust paradox.” While the general perception of the tool was positive, a significant gap was observed in autonomous confidence. Only 23% of participants agreed that they did not require additional explanations to trust the AI model’s output. This finding highlights the persistence of the black box problem. Despite the technical accuracy of the models, clinicians demand transparency regarding why a decision is made, especially when the algorithm contradicts their expert intuition.

### Clinical variability and the nature of the diagnostic task

The data suggests that AI models addressing tasks with a clear anatomical ground truth enjoy more immediate acceptance. For instance, the detection of mesorectal fascia invasion in rectal cancer, which can be validated *via* MRI and subsequent pathology, showed higher levels of clinical integration. In contrast, abstract prognostic tasks, such as the estimation of overall survival in lung cancer, generated greater skepticism.

This discrepancy likely arises because survival is a multifactorial outcome dependent on genomics, treatment, and comorbidities, making it difficult to deduce solely from an initial image. When a model provides a prediction without justifying it through integrated multimodal data, it creates dissonance for the clinicians. This heterogeneity implies that there is no universal approach to AI validation in oncology. Platforms must be inherently adaptable, offering different visualization modes and varying levels of contextual information tailored to the biological and diagnostic complexity of each specific cancer type.

### Limitations and study challenges

Despite the advancements presented, this study has certain limitations. Following the ISCT credibility assessment principles, we have categorized the potential biases of this validation process into three distinct levels [31]:

- **Data level:** The research relies on retrospective data within an *in silico* clinical trial framework. While the curated datasets from the CHAIMELEON project are of high quality, they may carry a selection bias that overestimates model performance compared to unselected, real-world populations. Furthermore, using retrospective cohorts does not fully replicate the complexity and environmental noise of prospective clinical settings.
- **Device level:** The platform’s capacity to provide transparency is dependent on the underlying AI model. If the algorithm does not produce native explainability outputs, such as attention maps or scores for feature importance, the system cannot visualize them, highlighting a persistent ‘black box’ limitation at the device level.
- **User level:** Although the three-stage workflow was designed to break potential automation bias and a 30-day washout period was enforced to mitigate recall bias, clinicians in an experimental setting might not experience the same cognitive fatigue or time pressure as in routine practice. This limitation regarding ‘ecological validity’ could lead to an overestimation of the tool’s usability or performance.

Finally, while the architecture is designed to be scalable, the current implementation requires substantial technical infrastructure, such as Kubernetes servers and database management. These requirements could represent a barrier to adoption for centers with limited IT resources, although the use of cloud solutions partially mitigates this concern. In fact, the primary goal of migrating this *in silico* validation platform from the CHAIMELEON project to the EUCAIM cloud is to ensure accessibility for all users within the network without the need for local installations.

## Supplementary information

**Additional File 1: Figure S1.** Customized user interfaces for Stage 1 (Clinical Evaluation) and Stage 2 (AI Trust Assessment) across lung, breast, colon, and rectum cancer cohorts.

## Data Availability

The datasets used for validating the platform were provided within the CHAIMELEON project. Long-term sustainability and access to both the platform and associated datasets are ensured through the EUCAIM infrastructure. Data can be requested by application *via* the EUCAIM dashboard https://dashboard.eucaim.cancerimage.eu/.
