# Supplementary material for: Bridging the AI chasm in oncology: a standardized platform to enable the in silico clinical validation of AI models within the CHAIMELEON Project
Source: Eur Radiol Exp. 2026 Jul 31;10:111. doi: 10.1186/s41747-026-00770-7 (PMC13427685; doi:10.1186/s41747-026-00770-7)

Bridging the AI chasm in oncology: a standardized platform to enable the *in silico* clinical validation of AI models within the CHAIMELEON Project

ELECTRONIC SUPPLEMENTARY MATERIAL

Supplementary Figure 1. Customized user interfaces for Stage 1 (Clinical Evaluation) and Stage 2 (AI Trust Assessment) across lung, breast, colon, and rectum cancer cohorts.

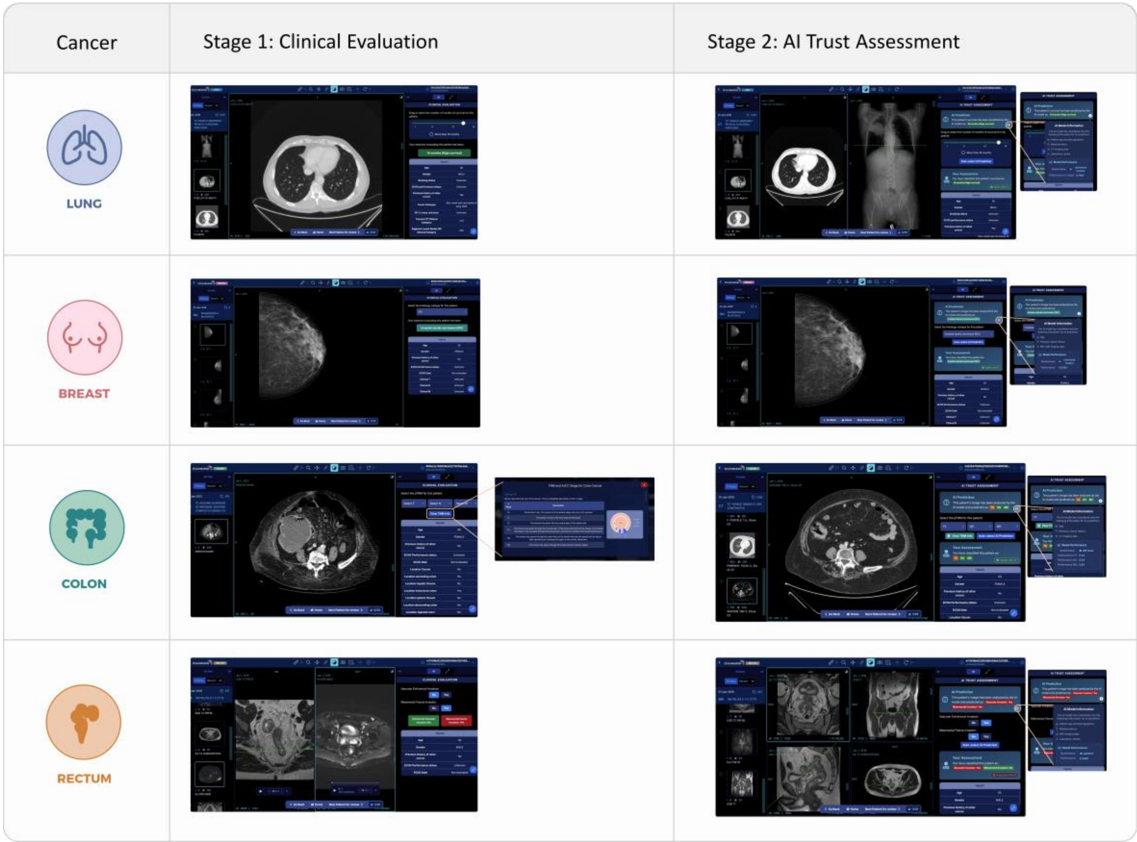

Supplement: Supplementary file 1 — Additional File 1: Figure S1. Customized user interfaces for Stage 1 (Clinical Evaluation) and Stage 2 (AI Trust Assessment) across lung, breast, colon, and rectum cancer cohorts. [file 41747_2026_770_MOESM1_ESM.pdf]
